# The association between umbilical cord blood fat-soluble vitamin concentrations and infant birth weight

**DOI:** 10.3389/fendo.2023.1048615

**Published:** 2023-09-21

**Authors:** Guicun Yang, Nianrong wang, Hao Liu, Lina Si, Yan Zhao

**Affiliations:** ^1^ Department of Pediatrics, Chongqing Health Center for Women and Children, Chongqing, China; ^2^ Department of Pediatrics, Women and Children’s Hospital of Chongqing Medical University, Chongqing, China

**Keywords:** vitamin A, vitamin D, vitamin E, fat-soluble vitamin, umbilical cord blood, birth weight

## Abstract

**Background:**

Fat-soluble vitamins, including vitamins A, D and E, play an important role in the regulation of glucose and lipid metabolism, and may affect infant birth weight. Evidence on the association of birthweight with fat-soluble vitamins is controversial. Therefore, this study aims is to determine the associations of birthweight with vitamin A, D, and E concentrations in cord blood.

**Methods:**

A total of 199 mother–infant pairs were enrolled in the study. According to gestational age and birth weight, the mother–infant pairs were divided into small for gestational age (SGA), appropriate for gestational age (AGA), and large for gestational age (LGA). The Vitamin A, D, and E concentrations in serum were measured by high-performance liquid chromatography tandem-mass spectrometry.

**Results:**

The concentrations of vitamin A in the SGA group were significantly lower than those in the AGA and LGA groups. The concentrations of vitamin E in the SGA group were significantly higher than those in the AGA and LGA groups. However, no significant differences were observed in vitamin D among the three groups. Being male (β = 0.317, p < 0.001) and birth weight (β = 0.229, p = 0.014) were positively correlated with the levels of vitamin A. Birth weight (β = -0.213, p= 0.026) was correlated with lower levels of vitamin E. No correlation was found between influencing Factors and the levels of vitamin D (p> 0.05). After adjusting for gestational age, sex, mother’s age, delivery mode, pre-pregnancy BMI, and weight gain during pregnancy, the levels of cord blood vitamin A were positively correlated with birth weight (p=0.012).

**Conclusion:**

The infant’s birth weight is associated with the levels of cord blood vitamins A and E. The dysregulation of vitamins A and E in infants may be a risk factor for fetal growth and future metabolic diseases.

## 1 Introduction

Birth weight (BW) is important to the health of the fetus, and it also predicts the subsequent development of the child. There are two types of extreme birth weight according to the gestational age and sex-specific (1): large for gestational age (LGA) and small for gestational age (SGA), and both of them increase the risk of cardiovascular disease, diabetes, and obesity later in life (2, 3). However, the mechanisms underlying the extreme birth weight and subsequent metabolic dysfunction are not well understood.

Maternal nutrition is important for fetal development and maternal health. Fat-soluble vitamins, including vitamins A, D and E, play an essential role in the regulation of glucose and lipid metabolism. Vitamin A and Vitamin D can affect obesity, cardiovascular disease, insulin resistance and type 2 diabetes (4, 5). While recent literature has found new mechanistic insights into the vitamin E derivatives in cardiovascular disease regulation (6). Vitamin E improves lipid metabolism in mice with nonalcoholic fatty liver through Nrf2/CES1 signaling pathway (7). Optimal maternal nutrition is a major factor in regulating fetal development and its lifelong consequences (8). The cord blood concentration of fat-soluble vitamins may influence the intrauterine environment, and may be associated with birth weight and lifelong metabolic dysfunction.

Evidence on the association of birthweight with fat-soluble vitamins is controversial. Recent studies have shown that premature low birth weight (LBW) infants have significantly lower cord blood vitamin A levels, and vitamin A levels in LBW infants correlate with their birth weight (9). However, Enfu Tao et al. found that cord blood vitamin A concentration in late preterm infants was not associated with birth weight (10). Some meta-analysis relating to vitamin revealed that maternal vitamin D deficiency increased the risk of SGA (OR=1.588, 95% CI 1.138-2.216) (11) and LBW (OR=2.39, 95% CI 1.25-4.57) (12). However, in the current literature, the difference in neonatal anthropometric measurements was not found between infants born to normal and vitamin D deficient mothers (13). Vitamin E was found to be positively associated with birth weight and reduced the risk of SGA (14). And one finding had been reported that high levels of vitamin E were associated with macrosomia (15).

Understanding fat-soluble vitamins in low and high birth-weight infants may provide new insights into metabolic mechanisms in newborns and help prevent subsequent metabolic diseases. This study aims to determine associations between birthweight and vitamin A, E, and D concentrations in cord blood.

## 2 Methods

### 2.1 Study population

Cord blood samples were collected from 199 newborns in the Department of Obstetrics of Chongqing Maternal and Child Health Hospital between June 2021 and December 2021. Inclusion criteria were singleton pregnancy, live, term birth (37–42 weeks), aged 18–42 years, no smoking and drinking history. Women with pregnancy complications, such as gestational hypertension, preeclampsia, cancer, or asthma were excluded. According to birth weight and gestational age (GA), a total of 199 mother–infant pairs were divided into SGA group (63), AGA (appropriate for gestational age) group (87), LGA group (49). Informed written consent was obtained. This study was approved by the Ethics Committee of our hospital.

### 2.2 Data collection

Data were collected from our hospital’s electronic information system, including pre-pregnancy weight, maternal height, gestational weight gain, sociodemographic information, delivery and neonatal outcomes (GA, newborn sex, birth weight, birth length). Baby weight and birth length were measured immediately after birth. Neonatal cord blood was collected during delivery. As soon as the samples were collected, they were sent without delay to the hospital laboratory, centrifuged, and frozen at -80°C until fat-soluble vitamin levels were analyzed.

### 2.3 Vitamin A, D and E measurement

The samples were analyzed at the Neonatal Screening Center, Chongqing Health Center for Women and Children. The Vitamin A, D and E concentrations in serum were measured by high-performance liquid chromatography tandem-mass spectrometry (HPLCMS/MS) using a Waters UPLC Xevo TQS (Waters Corporation, Milford, MA, USA) described by Hao Liu et al. (16). Briefly, QCs, standards, and plasma samples were added into the corresponding wells of 96-wells plates. Protein was precipitated by adding isotopic IS in a 35:1 (v/v) mixture of methanol: isopropanol, then hexane was added to each well for extraction. The Vitamin A, D and E in serum was separated by HPLC on a Shimadzu Waters BEH C18μm 1.7 2.1*50mm column and quantitated by MS.

### 2.4 Variables and definitions

Birth weight was measured immediately after delivery by midwives using electronic scales to the nearest 10 g. According to the birth weight curve of newborns in China, infants with birth weight less than the 10th percentile and greater than the 90th percentile for gestational age and sex were divided into SGA and LGA (17). Prepregnancy BMI was calculated by dividing self-reported weight in kilograms by height in meters squared. Gestational weight gain was calculated from prenatal weight and pre-pregnancy weight. Babies born between 37-42 weeks of pregnancy were defined as term birth. The GA was confirmed through the use of ultrasound before week 20 of gestation.

### 2.5 Statistical analysis

All analyses were done with SPSS version 25.0. Data are presented as mean ± standard deviation (SD) for continuous variables, medians and interquartile range for skewed distributions, and counts (percentages) for categorical variables. Comparisons among subjects in the SGA, AGA, and LGA groups were made using One-way ANOVA. Multivariate linear regression models were used to estimate the influencing factors on vitamins A, D, and E in cord blood and birth weight after adjusting for confounders. Associations between fat-soluble vitamins and neonatal birth weight adjusted for confounders were evaluated by partial correlation analysis. The confounders considered were mode of delivery, GA, maternal age, pre-pregnancy BMI, and sex of the infants. A p-value <0.05 was considered statistically significant.

## 3 Results

### 3.1 Baseline characteristics of the mother–infant pairs

**Table 1** shows baseline characteristics of mothers and birth outcomes. This study enrolled a total of 199 mother-infant pairs, including 63 in the SGA group, 87 in the AGA group and 49 in the LGA group. Statistically significant differences were found in maternal age (*P*=0.005), delivery mode (*P*<0.001), gestational age (*P*=0.005), birth weight (*P*<0.001), birth length (*P*<0.001), and weight gain (*P*<0.001) among the three groups. Women with LGA babies were less likely to deliver spontaneously, and have higher weight gain during pregnancy and prepregnancy BMI than SGA and AGA groups.

**Table 1:** Baseline characteristics of the mother–infant pairs in the three groups.

	Total(n=199)	SGA(n=63)	AGA(n=87)	LGA(n=49)	*P*-value
Maternal information					
Maternal age (years)	29.9 ± 3.8	28.9 ± 3.8	30.6 ± 3.8	29.4 ± 3.8	0.005*
Delivery mode (SVD/CS), n (%)	100(50.2%)/99(49.8%)	48(76.2%)/15(23.8%)	37(42.5%)/50(57.5%)	15(30.6%)/34(69.4%)	<0.001***
Prepregnancy maternal body mass index, mean ± SD	21.2 ± 2.9	20.3 ± 2.5	21.0 ± 2.8	22.6 ± 3.3	<0.001***
Weight gain during pregnancy	13.2 ± 4.4	12.3 ± 4.3	12.7 ± 4.4	15.5 ± 3.9	<0.001***
Neonatal information					
Gestational age (weeks)	38.8 ± 1.06	38.7 ± 1.1	38.7 ± 1.0	39.2 ± 0.9	0.005*
Gender (male/female), n (%)	96(48.2%)/103(51.8%)	34(54%)/29(46%)	35(40%)/52(60%)	27(55%)/22(45%)	0.136
Birth weight (g)	3120(2750,3760)	2650(2480,2790)	3200(2930,3420)	4010(3870,4265)	<0.001***
Birth length (cm)	49.4 ± 2.2	47.4 ± 1.6	49.4 ± 1.3	51.9 ± 1.3	<0.001***
Cord blood metabolic measures					
vitamin A (ng/ml)	229.6 ± 67.4	214.3 ± 58.1	226.0 ± 65.5	255.5 ± 75.3	0.004*
vitamin D (ng/ml)	19.7 ± 8.0	19.9 ± 8.8	19.2 ± 8.0	20.3 ± 7.2	0.711
vitamin E (ng/ml)	2196.0 ± 600.6	2388.0 ± 649.9	2069.6 ± 516.2	2176.2 ± 622.6	0.005*

Data presented are medians, mean ± SD for continuous variables, n (%) for categorical variables, and interquartile range for skewed distributions. The p-values are from One-way ANOVA for differences in means for continuous variables or chi-square tests for differences in proportions for categorical variables groups. Values in bold are significant at p < 0.05.SVD, spontaneous vaginal delivery; CS, cesarean section. *p < 0.05, ***p < 0.001.

### 3.2 Comparisons of fat-soluble vitamins

Statistically significant differences were found in cord blood vitamin A and E concentrations among the groups; No significant differences were observed in vitamin D. The concentrations of vitamin A in the SGA group were significantly lower than those in the AGA and LGA group; however, the concentrations of vitamin E in the SGA group were significantly higher than those in the AGA and LGA group (**Table 1**).

### 3.3 Analysis of the influencing factors on birth weight

GA, spontaneous vaginal delivery (SVD), weight gain during pregnancy, pre-pregnancy BMI, Vitamin A, and Vitamin E were associated with birth weight. GA (β = 0.480, *p*<0.001), Pre-pregnancy BMI (β = 0.260, p<0.001), Weight gain during pregnancy (β = 0.248, *p*<0.001), Vitamin A (β = 0.146, *p*=0.009) and being SVD (β = 0.277, *p*<0.001) were positively correlated with the birth weight, while Vitamin E (β = −0.135, *p* = 0.015) negatively correlated with the birth weight (**Table 2**).

**Table 2:** Multivariate linear regression analysis of the influencing factors on birth weight.

	β	95%IC		p-value
Maternal age	0.058	0.285	-7.394	0.285
Gestational age	0.480	207.516	323.76	<0.001***
Gender (male = 1)	-0.092	-236.166	19.283	0.096
SVD (yes = 1)	0.277	193.034	455.842	<0.001***
Pre-pregnancy BMI (kg/m2)	0.260	29.676	73.567	<0.001***
Weight gain during pregnancy	0.258	19.567	49.513	<0.001***
Vitamin A	0.146	0.320	2.230	0.009*
Vitamin D	0.011	-7.117	8.711	0.843
Vitamin E	-0.135	-0.283	-0.125	0.015*

Multivariate linear regression analysis was used with adjustments for maternal age, mode of delivery, gestational age, sex, pre-pregnancy BMI, weight gain during pregnancy and fat-soluble vitamins. Values in bold are significant at p <0.05.SVD, spontaneous vaginal delivery; BMI, body mass index. *p < 0.05, ***p < 0.001.

### 3.4 Analysis of factors of fat-soluble vitamins

Sex and birth weight were associated with the levels of vitamin A in umbilical cord blood. Being male (β = 0.317, *p* < 0.001) and birth weight (β = 0.229, *p* = 0.014) were positively correlated with the levels of vitamin A. Birth weight (β = -0.213, *p*= 0.026) was correlated with lower levels of vitamin E. No correlation was found between influencing Factors and the levels of vitamin D (*p*> 0.05) (**Table 3**).

**Table 3:** Multivariate linear regression analysis of the influencing factors for the levels vitamin A, vitamin D, and vitamin E in cord blood.

	β	95%IC		p-value
Vitamin A				
Maternal age	-0.50	-3.304	1.543	0.474
Gestational age	-0.86	-15.712	4.834	0.298
Gender (male=1)	0.317	24.416	60.894	<0.001***
SVD (yes = 1)	-0.148	-40.421	0.520	0.056
Pre-pregnancy BMI (kg/m2)	0.048	-2.362	4.535	0.535
Weight gain during pregnancy	0.03	-1.875	2.785	0.701
Birth weight	0.229	0.005	0.047	0.014*
Vitamin D				
Maternal age	0.004	-0.300	0.315	0.961
Gestational age	-0.009	-1.372	1.236	0.918
Gender (male = 1)	0.077	-1.086	3.544	0.296
SVD (yes = 1)	0.050	-1.799	3.397	0.545
Pre-pregnancy BMI (kg/m2)	-0.077	-0.645	0.23	0.950
Weight gain during pregnancy	-0.068	-0.42	0.172	0.409
Birth weight	<0.001	-0.003	0.003	1
Vitamin E				
Maternal age	-0.034	-27.557	17.037	0.643
Gestational age	0.144	-13.074	176.178	0.091
Gender (male = 1)	0.002	-165.384	170.621	0.976
SVD (yes = 1)	-0.118	-330.224	44.896	0.140
Pre-pregnancy BMI (kg/m2)	0.021	27.428	360.097	0.788
Weight gain during pregnancy	0.133	-3.277	39.649	0.096
Birth weight	-0.213	-0.409	-0.026	0.026*

Multivariate linear regression analysis was used with adjustments for maternal age, mode of delivery, gestational age, sex, pre-pregnancy BMI, birth weight and weight gain during pregnancy. Values in bold are significant at p <0.05.SVD, spontaneous vaginal delivery; BMI, body mass index. *p < 0.05, ***p < 0.001.

### 3.5 Partial correlations between fat-soluble vitamins and newborn birth weight

The partial correlation coefficients between fat-soluble vitamins and newborn birth weight are shown in **Table 4**. After adjusting for delivery mode, GA, mother’s age, sex, pre-pregnancy BMI, pregnancy weight gain and other factors, the cord blood vitamin A level i was positively correlated with body weight (p=0.012). However, no statistically significant association was found between vitamin D or vitamin E level and birth weight.

**Table 4:** Relationship between the levels of vitamins A, D and E and infant birth weight.

	vitamins A		vitamins D		vitamins E	
	*r*	*p-value*	*r*	*p-value*	*r*	*p-value*
Birth weight (g)	0.181	0.012	0.016	0.829	-0.140	0.053

Partial correlation analysis was used with adjustments for maternal age, mode of delivery, gestational age, gender, pre-pregnancy BMI, and weight gain during pregnancy. r, partial correlation coefficient; BMI, body mass index.

## 4 Discussion

Our study demonstrates the relationship between the levels of cord blood fat-soluble vitamins and the BW. We found that the cord blood vitamin A level was positively correlated with the infant BW, while the vitamin E level was negatively correlated with BW. When compared with those of AGA and LGA, the vitamin A level was substantially lower in SGA, whereas the vitamin E level was significantly higher. However, we found no significant association between cord blood vitamin D serum level with birth weight.

Vitamin A is critical to ensure proper embryonic development and is involved in several metabolic pathways (18). Observational studies have found that cord blood and maternal vitamin A levels were significantly correlated with birth weight and length (19–22). The largest study investigating untargeted metabolic profiles showed that cord blood vitamin A levels were associated with birthweight (23), after controlling for hereditary factors such as parental size. Indeed, the vitamin A supplementation study in Human Immunodeficiency Virus–Infected Women have indicated that maternal vitamin A supplementation increase birth weight and neonatal growth, and decreases anemia (24). In our study, we found that the cord blood vitamin A concentrations were associated with birth weight, which is consistent with previous research findings. This finding suggests that there might be a role of vitamin A in determining birth weight and underscores the importance of vitamin A nutrition in fetal growth. However, some studies have shown that maternal vitamin A concentrations were not associated with birth weight (10, 25). The reasons behind these discrepancies might be partly due to different study participants. In our study, the Mother–Infant Pairs were all healthy and born full term, therefore, the effects of premature birth, infection, and maternal disease on vitamin A were excluded.

Here, we also discovered that SGA infants exhibit lower concentrations of cord blood vitamin A compared to AGA infants. As some studies have found that umbilical cord blood vitamin A serum concentration is significantly correlated with maternal vitamin A status (21, 26), SGA infants may be born from vitamin A deficient mothers. Unfortunately, we did not measure serum vitamin A levels in pregnant mothers. We further observed that LGA infants had higher concentrations of cord blood vitamin A compared with AGA infants. As maternal nutritional status has a key role on fetal growth (27), this result suggested that maintaining a high concentration of vitamin A during pregnancy may not be good for fetal growth.

In this study, we found no significant association between cord blood vitamin D serum levels with birth weight, which does not agree with the findings of previous studies (11, 12, 28). A meta-analysis of observational studies has shown that vitamin D deficiency during pregnancy is associated with a higher risk of low birth weight (12). While a Brazilian cohort study found that higher concentrations of vitamin D during the first, second, and third trimesters increase the risk of preterm birth (28). A meta-analysis has suggested that vitamin D deficiency is associated with an increased risk of SGA (11). However, in our study, we found no association between cord blood vitamin D levels and SGA. Three limitations may have biased the result, particularly the fact that our study subjects were full-term infants, excluding preterm infants. The second limitation the plasma we tested came from cord blood, which does not signify the entire course of pregnancy. The third limitation was the relatively small sample size. In light of the conflicting data from studies, larger-sample studies are required to further confirm the effects of vitamin D on birth weight.

Vitamin E is a regulator of glucose and lipid metabolism and vitamin E deficiency causes metabolic dysregulation in the early stages of development. Therefore, cord blood vitamin E serum levels may influence infant birth weight. Several studies have demonstrated that vitamin E deficiency was found in VLBW infants (29, 30). While a Chinese observational study has indicated that vitamin E concentrations were positively related to increased fetal growth, and a positive association between vitamin E and macrosomia (15). In our study, we found that vitamin E was inversely related to birth weight. The results of this study indicate that the findings are different from what has been previously reported by other researchers. The possible influence factors were the different gestational ages of study participants and the source of the blood. And in another study, vitamin E concentrations were related to decreased risk of SGA births, and an increased risk of LGA (14). Here, we found that cord blood vitamin E concentrations were significantly higher in SGA than those in AGA and LGA. These findings suggest that vitamin E levels may affect on birth weight, but it is still controversial. Further studies are needed to investigate the relationship and mechanism between vitamin E and birth weight.

The strengths of this research include not only comparing the levels of fat-soluble vitamins in umbilical cord blood, but also dividing infants into subgroups based on their GA and BW. One limitation of the present study is the small number of participants from only one hospital in China, thus, it may not be sufficient in power to be generalizable. In the future, a large number of multicenter studies are needed.

In conclusion, this study found that a lower concentration of vitamin A in cord blood was associated with a higher risk of SGA and a lower risk of LGA. Additionally, excessive concentration of vitamin E in cord blood was found to be associated with SGA. Since the vitamin status of newborns is mostly dependent on that of their mothers, women are recommended to avoid having high concentrations of vitamin A and E during pregnancy. Nonetheless, further prospective studies are needed to explore these associations in different populations and to understand the mechanism linking vitamin A and E to infant birth weight.

## Data availability statement

The raw data supporting the conclusions of this article will be made available by the authors, without undue reservation.

## Ethics statement

The studies involving human participants were reviewed and approved by the ethics committee of Chongqing Health Center for Women and Children. The patients/participants provided their written informed consent to participate in this study.

## Author contributions

YZ and GY conceived the study. GY performed the data analysis and wrote the manuscript. HL contributed to manuscript preparation. NW and LS helped with data analysis. All the authors contributed to the manuscript and approved the submitted version.
